# Supplementary material for: MetaQTL: a package of new computational methods for the meta-analysis of QTL mapping experiments
Source: BMC Bioinformatics. 2007 Feb 8;8:49. doi: 10.1186/1471-2105-8-49 (PMC1808479; doi:10.1186/1471-2105-8-49)
Supplement: Additional File 7 — MetaQTL Package : jar file and tutorial. This Zip archive contains both the MetaQTL JAR file and the files of the tutorial. [file 1471-2105-8-49-S7.zip › org.inra.metaqtl/doc/org/metaqtl/class-use/ChromCluster.html]

Uses of Class org.metaqtl.ChromCluster


|  |  |  |
| --- | --- | --- |
| **Overview** \| **Package** \| **Class** \| **Use** \| **Tree** \| **Deprecated** \| **Index** \| **Help** | |  |
| PREV   NEXT | **FRAMES**    **NO FRAMES**     **All Classes** |  |


---


## **Uses of Class org.metaqtl.ChromCluster**

| Packages that use ChromCluster | |
| --- | --- |
| **org.metaqtl** |  |
| **org.metaqtl.algo** |  |
| **org.metaqtl.main** |  |

Uses of ChromCluster in org.metaqtl

| Fields in org.metaqtl declared as ChromCluster | |
| --- | --- |
| `ChromCluster` | `ChromCluster.ClusterGraph.cluster` |

| Methods in org.metaqtl that return ChromCluster | |
| --- | --- |
| `ChromCluster[]` | `MapCluster.getClusters()`             Returns the chromosome clusters. |

Constructors in org.metaqtl with parameters of type ChromCluster

`ChromCluster.ClusterGraph(ChromCluster cluster)`

`MetaChrom(MultiFitResult result,
ChromCluster chromCluster)`
  
          Get a new instance of `MetaChrom` which is
initialized by using the result
of the merge of the members of a `ChromCluster`.

Uses of ChromCluster in org.metaqtl.algo

| Methods in org.metaqtl.algo with parameters of type ChromCluster | |
| --- | --- |
| `protected  MetaChrom` | `MetaMapAlgorithm.buildMetaChrom(ChromCluster cluster)`             For a cluster of chromosomes `cluster` this method builds the consensus chromosome using a Weighted Least Square strategy and returns the result as a `MetaChrom` object. |
| `void` | `QtlProjAlgorithm.checkProjection(ChromCluster[] chromClusters)` |
| `void` | `QtlProjAlgorithm.doProjection(ChromCluster[] chromClusters)` |

Uses of ChromCluster in org.metaqtl.main

| Methods in org.metaqtl.main that return ChromCluster | |
| --- | --- |
| `static ChromCluster[]` | `MetaMain.getClusterByNames(ChromCluster[] clusters, java.lang.String[] chromNames)` |

| Methods in org.metaqtl.main with parameters of type ChromCluster | |
| --- | --- |
| `static ChromCluster[]` | `MetaMain.getClusterByNames(ChromCluster[] clusters, java.lang.String[] chromNames)` |

---


|  |  |  |
| --- | --- | --- |
| **Overview** \| **Package** \| **Class** \| **Use** \| **Tree** \| **Deprecated** \| **Index** \| **Help** | |  |
| PREV   NEXT | **FRAMES**    **NO FRAMES**     **All Classes** |  |


---
